# Targeted Deletion of Fibrinogen Like Protein 1 Reveals a Novel Role in Energy Substrate Utilization

**DOI:** 10.1371/journal.pone.0058084

**Published:** 2013-03-06

**Authors:** Valeriy Demchev, Geraldine Malana, Divya Vangala, Janis Stoll, Anal Desai, Hye Won Kang, Yingxia Li, Hamed Nayeb-Hashemi, Michele Niepel, David E. Cohen, Chinweike Ukomadu

**Affiliations:** Division of Gastroenterology, Department of Medicine, Brigham and Women’s Hospital, Harvard Medical School, Boston, Massachusetts, United States of America; University of Santiago de Compostela School of Medicine - CIMUS, Spain

## Abstract

Fibrinogen like protein 1(Fgl1) is a secreted protein with mitogenic activity on primary hepatocytes. Fgl1 is expressed in the liver and its expression is enhanced following acute liver injury. In animals with acute liver failure, administration of recombinant Fgl1 results in decreased mortality supporting the notion that Fgl1 stimulates hepatocyte proliferation and/or protects hepatocytes from injury. However, because Fgl1 is secreted and detected in the plasma, it is possible that the role of Fgl1 extends far beyond its effect on hepatocytes. In this study, we show that Fgl1 is additionally expressed in brown adipose tissue. We find that signals elaborated following liver injury also enhance the expression of Fgl1 in brown adipose tissue suggesting that there is a cross talk between the injured liver and adipose tissues. To identify extra hepatic effects, we generated Fgl1 deficient mice. These mice exhibit a phenotype suggestive of a global metabolic defect: Fgl1 null mice are heavier than wild type mates, have abnormal plasma lipid profiles, fasting hyperglycemia with enhanced gluconeogenesis and exhibit differences in white and brown adipose tissue morphology when compared to wild types. Because Fgl1 shares structural similarity to Angiopoietin like factors 2, 3, 4 and 6 which regulate lipid metabolism and energy utilization, we postulate that Fgl1 is a member of an emerging group of proteins with key roles in metabolism and liver regeneration.

## Introduction

Fibrinogen like protein 1 (Fgl1 also called HRFEP-1, also called hepassocin) was initially identified as an overexpressed transcript in hepatocellular carcinoma [1] and as a transcript enriched in regenerating rodent livers [2]. Analysis of multiple human and rat tissues and human cell culture lines suggest that Fgl1 is mostly expressed in the liver or hepatocyte derived cell lines [1], [3]. Both human and rodent FGL1 contain a canonical signal recognition sequence and not surprisingly, the protein is detected in culture media of expressing cells and in the plasma of rodents and humans [3], [4], [5].

Recombinant Fgl1 stimulates tritiated thymidine uptake in rodent primary hepatocytes at a level similar to that of hepatocyte mitogens such as epidermal growth factor [3]. In addition, administration of purified recombinant Fgl1 to rodents with toxin induced fulminant liver failure reduces mortality [6]. Although there is a basal level of Fgl1 expression in rodent livers, there is marked induction of expression after liver injury as occurs with 70% partial hepatectomy (PH) [2], [7], which parallels the injury mediated elevation in IL-6 [8]. Indeed, treatment of Hep G2 cells with IL-6 leads to robust expression and subsequent enhanced secretion into the culture media [4] and either the sub-cutaneous injection of turpentine (an inducer of IL-6) or the intraperitoneal injection of recombinant IL-6 into rodents leads to enhanced plasma levels of Fgl1 ([4], [9] and CU, unpublished). In addition to IL-6, recent studies have shown that hepatocyte nuclear factor 1-alpha (HNF1α), regulates the expression of Fgl1 *in vivo* and that deletion of a specific HNF1α site abrogates Fgl1 promoter activation [9]. Taken together, these studies suggest that Fgl1 is a liver secreted factor that acts in an autocrine/paracrine fashion to enhance hepatocyte proliferation and/or protect hepatocytes from injury.

Fgl1 is structurally similar to Angiopoietin like factors (ANGPTLs). It contains an N-terminal signal recognition peptide, a potential N-terminal coil-coil domain, a C-terminal fibrinogen related domain (FReD) and multiple cysteines presumably used for inter and intra molecular disulfide bonds. Recent data suggest that a number of ANGPTL proteins, specifically ANGPTL 2, 3, 4 and 6 play regulatory roles in lipid and energy metabolism. For example, mice lacking *Angptl3 or Angptl4* have low plasma triglyceride and cholesterol concentrations [10], [11], [12]. Multiple lines of evidence show that Angptl3 and Angptl4 regulate plasma triglyceride levels through the inhibition of lipoprotein lipase and/or hepatic lipase [11], [13], [14] and/or through stimulation of lipolysis in adipose tissue [15], [16]. The deletion of *Angptl2* in mice leads to improvement in diet induced obesity and insulin resistance [17]. Lastly, *Angptl6^−/−^* mice are obese, hyperinsulinemic and accumulate significant amounts of triglycerides in the liver and skeletal muscle [18], whereas transgenic mice that overexpress Angptl6 in the liver, remain lean on a high fat diet [18]. The structural similarity between these proteins and Fgl1 raises the intriguing possibility that Fgl1 may not only regulate liver proliferative processes but may also play a role in lipid metabolism and energy utilization.

In this study we show that Fgl1 is expressed not only in liver but also adipose tissues. Remarkably, expression in brown adipose tissue is enhanced following 70% PH in mice. We generated an Fgl1 knock out mouse and find that it is larger than age matched wild type control mice, exhibits fasting hyperglycemia, changes in lipid metabolism, structural defects in brown and white adipose tissues and impaired expression of brown and white fat genes. We conclude that Fgl1 is a liver and adipose tissue regulatory factor and that it may serve as novel factor to interrogate the link between adipocyte function and hepatocyte proliferation.

## Methods

### The *Fgl1* Null Mouse

The *Fgl1* gene contains 8 exons spanning over 24 kilobases (kb)of genomic DNA. The initiator ATG resides in the second exon. To disrupt *Fgl1*, we replaced exons 2 and 3 with a lox-neo-lox (LNL) cassette which removes the initiator ATG and abolishes the secretory signal recognition peptide. The resulting targeting vector was electroporated into PTL1 (129B6) hybrid ES cells. Several ES cells containing the targeting construct were identified by PCR. Male chimeras were bred to C57BL/6 females for germ-line transmission of the Fgl1 null allele. Mice heterozygous for the Fgl1 allele (*Fgl1^+/−^*) were then intercrossed to generate homozygous *Fgl1^−/−^* mice on a mixed 129/B6 genetic background. Animals were genotyped and allelic composition identified through the generation of a 215-bp (wild type) or 322-bp (disrupted allele) upon PCR amplification. Primers for genotyping were *Fgl1^+/+^*: forward 5′-AGCTGCCTGTGTTATTTCCTCTCA-3′ and reverse 5′-CCAGGGAGCCATTTTATTTATCCAA-3′. *Fgl1^−/−^* : forward 5′-ACCAGCCTCCTCTTAACAACTTTCT-3′ and reverse 5′-GGACGTAAA CTCCTCTTCAGACCTA-3′.

### Animals

Ethics Statement: All animal experiments were performed according to institutional guidelines of Harvard Medical School. All protocols with animal experimentation conformed to criteria outlined in the National Institutes of Health “Guide for the Care and Use of Laboratory Animals.” Protocols were reviewed and approved by the animal use committee of Harvard Medical School (protocol number 04193). Except where otherwise specified, the mice were fed normal chow diet (LabDiet, Brentwood, MO) with free access to food and water and were 3–4 months old males.

### Tissue Harvest and Surgery

Mice were weighed prior to sacrifice. Following euthanasia, liver, white adipose from the inguinal fat pads, brown adipose, cardiac and quadriceps skeletal muscle tissues were removed and weighed. In some instances the length of the animal from the nose to the anus was determined. For 70% PH, surgeries were performed on *Fgl1* ^+/+^ and *Fgl1^−/−^* mice as previously described [19] with the left and median lobes of the liver were removed. This tissue was used as the pre-partial hepatectomy sample. At 24 h and 48 h after PH, animals were euthanized; liver and brown adipose tissue was removed, immersed in 10% formalin or frozen in liquid nitrogen and stored at −80°C for later analysis. For determination of bromo deoxyuridine (BRDU) incorporation, animals were injected with 100 µg/kg of BRDU dissolved in sterile PBS by intraperitoneal (i.p.) route, 2 h prior to tissue harvest. Formalin fixed samples were then processed for immunohistochemistry. Of note, except where noted animals had free access to food and water prior to tissue harvest.

### Western Immunoblots

Briefly, liver tissue was homogenized in RIPA buffer (25 mM Hepes PH 7.4, 150 mM NaCl, 1% Triton X-100, 1% deoxycholic acid, 0.1% SDS, 2 mM EDTA) with a tissue grinder. The resulting homogenate was spun at 13,000 RPM. The supernatant was transferred to clean tubes, the protein concentration was determined and lysates were then denatured with Lammeli sample buffer. Approximately 60 µg of protein was loaded per lane on a 10% SDS-PAGE. Western blots were performed as previously described [20].

### Nucleic Acid Assays

PCR assays (qualitative and quantitative) were performed as previously described [21]. mRNA levels were determined relative to cyclophilin A. Table S1 in File S1 shows primer sequences used for these assays.

### Plasma Triglyceride, Cholesterol and Free Fatty Acid Concentrations

Plasma was obtained from mice wild type and null for Fgl1. Plasma triglyceride, cholesterol and free fatty acids were determined using reagents kits (Wako, Richmond, VA) as per manufacturer’s instruction. Fast protein liquid chromatography (FPLC) analysis was performed as previously reported [22].

### Glucose Tolerance Tests

*Fgl1^+/+^* and *Fgl1^−/−^* mice were fasted overnight (14–16 h) with free access to water. After obtaining baseline blood glucose levels each mouse received 1 mg/g of body weight of 20% glucose (Sigma, St Louis, MO) by i.p. injection. Blood glucose readings were then obtained 15, 30, 60, 90, 120, and 180 min after initial injection of glucose. After 180 min, mice were returned to their cages.

### Pyruvate Tolerance Tests

*Fgl1^+/+^* and *Fgl1^−/−^* mice were fasted overnight (14–16 h) with free access to water. After obtaining baseline blood glucose levels, each mouse received 2 g/kg of body weight i.p. injection of sodium pyruvate (Sigma, St. Louis, MO). Blood glucose readings were then obtained 15, 30, 60, 90, 120, and 180 min after initial injection of glucose. After 180 min mice were returned to their cages.

### Insulin Tolerance Tests

*Fgl1^+/+^* and *Fgl1^−/−^* mice were fasted for 16 h before testing. Baseline blood glucose levels were obtained then each mouse was injected with 1 U/kg of body weight of insulin (Eli Lilly, Indianapolis, IN). Blood glucose readings were obtained at 15, 30, 45 and 60 min after initial injection of insulin.

### Plasma Insulin Concentrations

Plasma insulin concentrations were determined by ELISA (Joslin Diabetes and Endocrinology Research Center Specialized Assay Core; supported in part by NIH 5P30 DK36836, Joslin Diabetes Center, Boston, MA, USA). Homeostatic Model of Assessment (HOMA) was determined using the formula: fasting glucose (mmol/L) × fasting insulin (mU/L)/22.5 [23].

### Measurement of Lipid Droplet Size

Lipid droplet area was measured with ImageJ software 1.46r (National Institutes of Health, Bethesda, MD). After appropriate thresholding, 40× magnification H/E images were converted to binary format. The watershed function was used to discriminate between individual droplets. Droplet size was measured with the droplet analysis function and area was converted to µm2 after determination of image scale. Data represented at mean droplet size+SEM. Differences between groups were assessed with non-paired Student’s t-test using Graphpad Prism statistical software (Graphpad Software Inc, La Jolla, CA).

### Food Consumption

3 month old mice wild type for (n = 4) and devoid of Fgl1 (n = 8) were housed individually and food intake measured daily. Prior to the onset of the experiments all cage clippings were removed and replaced with DACB liner paper (GQF manufacturing, Savannah, GA). Food was provided in a food cup and all animals had free access to water. Everyday for 18 days, total food consumed per day was determined by taking the difference between the weight of food placed in the cup and the weight of food remaining, including food particles on the ground. Animals were also weighed daily. Analysis of covariants was used to determine if there is any correlation between food intake and genotype.

### Indirect Calorimetry

*Fgl1^+/+^* and *Fgl1* ^−/−^ mice were individually housed in calorimetric chambers (Columbus Instruments, Columbus, OH) with free access to food and water. After a 24 h acclimation period, food consumption and gas exchange was determined. The rates of oxygen consumption (VO_2_, ml/kg/h) and of CO2 production (VCO_2_, ml/kg/h) were determined for individual mice and then used to determine day, night and total averages for each variable. The respiratory quotient (RER) was then calculated as the VCO_2_/VO_2_. Heat (kcal/h) was calculated as (caloric value (CV) ×VO_2_×weight (kg) where CV equals 3.815+ (1.232×RER)). Activity was determined by series of beam breaks per mouse and was calculated as average beam break per hour. VO_2_, VCO_2_, RER, heat and activity were calculated as light (0700 h –1900 h) and dark (1901 h –0659 h) and total (0700 h –0659 h) averages per mouse and then the *Fgl1^+/+^* and *Fgl1^−/−^* mice were averaged and reported as mean and SEM on graphs using Excel and Graphpad Prism 5.

### ^18^F-fluorodeoxyglucose Uptake

Mice were injected with 350 µCi ^18^F-fluorodeoxyglucose (^18^FDG). Animals were sacrificed and tissue samples of heart, muscle, white adipose and brown adipose were collected and washed with PBS. Tissues were weighed and radioactivity was measured using a Wallac Wizard Gamma Counter (GMI Inc, Ramsey, MN) to obtain the % injected dose per gram (%ID/g).

### Statistical Analysis

Data presented in the manuscript are means. Error bars represent SEM. For GTT, ITT and PTT tests, all data for the area under the curve analysis (AUC) were normalized to basal glucose levels of each mouse. Statistical significances was determined using two tailed unpaired student t test, one way and two way ANOVA on a Graphpad Prism 5. Mixed regression analysis was performed on SAS version 9.3 (SAS Institute, Cary, NC).

## Results

### Basal and Inducible Expression of Fgl1 in Adipose Tissue

**Figure 1:**
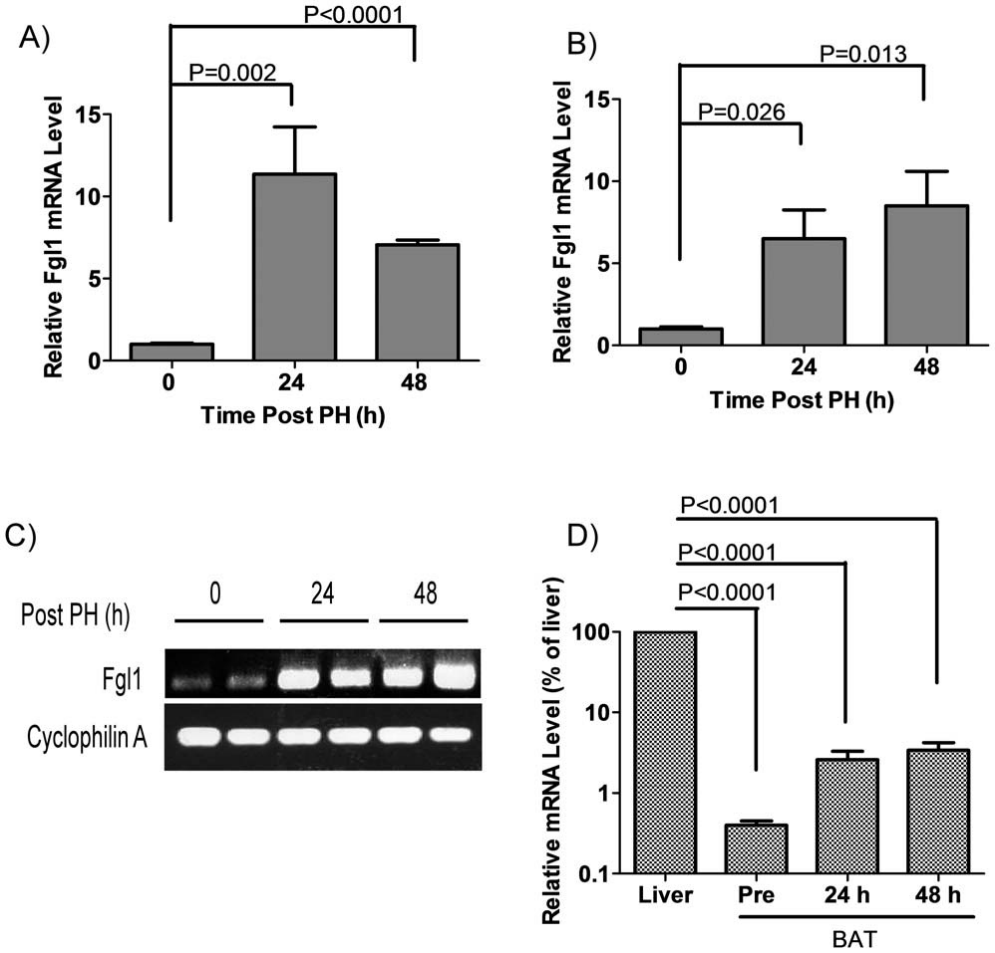
Fgl1 in hepatic and brown adipose tissue. A) mRNA levels of Fgl1 in the liver at baseline (0 h) and at 24 h and 48 h after PH. Note the increased expression after PH. P = 0.002 between 0 h and 24 h and less than 0.0001 between 0 h and 48 h after PH. The difference between levels at 24 h and 48 h is not significant. B) mRNA levels of Fgl1 in brown adipose tissue (BAT) at baseline and at 24 h and 48 h after partial hepatectomy. Fgl1 is detectable in BAT prior to injury but expression is enhanced after PH. P = 0.026 between 0 and 24 h and P = 0.013 between 0 and 48 h after PH. The difference between levels at 24 h and 48 h is not significant. n = 3 for samples in 1A and B. C) Gel electrophoresis of amplified cDNA from BAT at baseline and at 24 h and 48 h after PH. Cyclophilin A is the loading control. D) Comparison of BAT Fgl1 levels pre and post PH with hepatic Fgl1 levels at baseline. Samples are normalized to hepatic Fgl1 at 100%. Fgl1 in BAT is 0.4%, 2.6% and 3.4% of hepatic levels at baseline and at 24 h and 48 h after PH (P<0.0001).

An analysis of the expression of FGL1 in 50 distinct adult and fetal human tissues revealed liver-specific expression with the exception of low-level expression in the pancreas [3]. Another evaluation of 12 rat tissues and 9 human cancer cell lines showed that FGL1 was similarly liver-specific in its expression [1]. However, these studies did not include adipose tissue, which is of interest given the structural similarity with ANGPTL proteins, which include members that regulate lipid and energy metabolism. Consequently, we performed qPCR assays for Fgl1 mRNA in liver, brown and white adipose tissues of mice. As anticipated, Fgl1 was expressed in the liver, and was upregulated 11 fold at 24 h (P = 0.002) and 7 fold at 48 h (P<0.0001) following PH (Figure 1A). We also detected low levels of Fgl1 mRNA in brown adipose tissue (Figure 1B (left most bar) and 1C (lanes 1and 2)), and in white adipose tissue (Figure S2 in File S1, 2^nd^ bar from the left and the first three lanes of the inset, lower panel). Interestingly, Fgl1 mRNA is upregulated 6.5 fold at 24 h (P = 0.026) and 8.5 fold at 48 h (P = 0.013) in brown adipose tissue following PH (Figure 1B and C) suggesting that the injury response of Fgl1 in the liver is sensed in brown adipose tissue. Despite this upregulation of Fgl1, mRNA levels remain lower in brown adipose tissue pre and post partial hepatectomy (250, 38 and 29 fold lower at baseline, 24 h and 48 h after PH respectively, P<0.0001 at each time point) compared to basal levels in the liver (Figure 1D). These experiments show that that Fgl1 is expressed in adipose tissues and that signals elaborated during liver injury regulates its expression in both the liver and brown adipose tissue.

### *Fgl1* Null Mice are Heavier than Wild Type Controls

**Figure 2:**
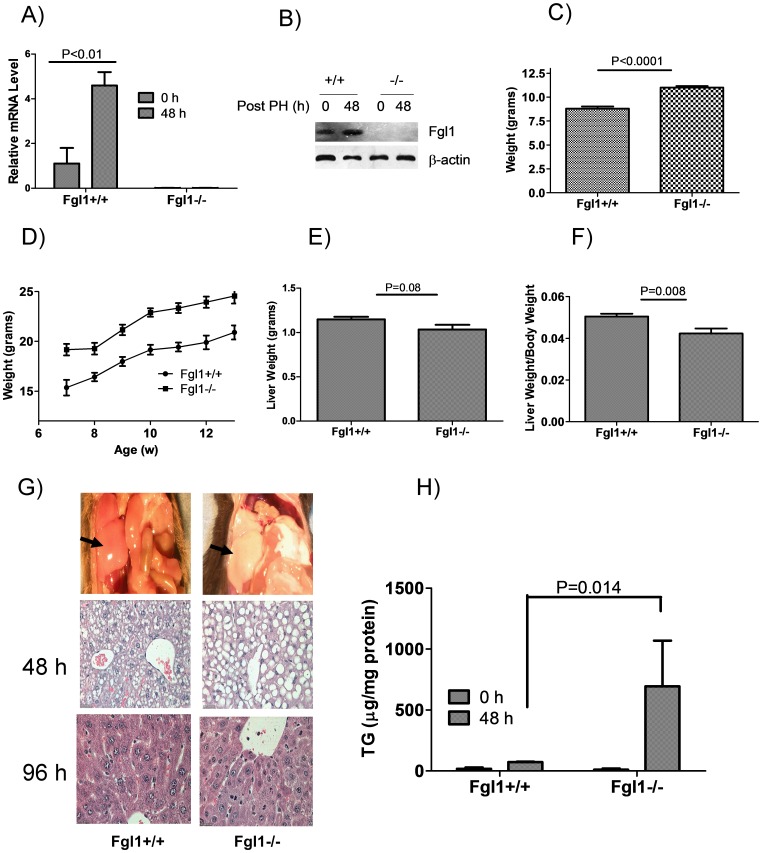
The liver in the Fgl1 knockout mouse. A) Fgl1 transcript is absent in the livers of the knockout mouse pre and post PH. Note the expected induction of Fgl1 in the wild type mouse after PH (P<0.01 for Fgl1 at baseline and 48 h after PH, n = 3 per group) B) Fgl1 protein is absent in the livers of the knockout mouse before and after PH while it is detectable at baseline and induced after PH in the wild type mouse. C) *Fgl1^−/−^* mice are larger than wild type mates as early as three weeks after birth (P<0.0001, n = 5 for each group). D) Representative graph of change in weight over time for *Fgl1^+/+^* (n = 8 for first 4 weeks, n = 4 for last three weeks) and *Fgl1^−/−^* (n = 9 for first 4 weeks and n = 8 for last three weeks). E) Liver mass is not different between *Fgl1^+/+^* and *Fgl1^−/−^* mice (n = 9 for each group). F) Liver weight to body weight ratio is smaller for *Fgl1^−/−^* mice (P = 0.008, n = 9). G) Marked lipid accumulation in the livers of *Fgl1^−/−^* mice after PH. Top: gross images of representative livers from *Fgl1^+/+^* (left) and *Fgl1^−/−^* (right). Arrows indicate remnant liver lobes. Middle: H&E images at 40× magnification of liver sections from *Fgl1^+/+^* and *Fgl1^−/−^* mice at 48 h post PH and bottom: similar H&E images at 96 h post PH. Note the resolution of steatosis in the *Fgl1^−/−^* mouse by 96 h after PH. H) Triglyceride (TG) content of liver extracts from *Fgl1^+/+^* and *Fgl1^−/−^* mice before and after PH. The difference between *Fgl1^−/−^* and *Fgl1^+/+^* at 48 h after PH is significant (P = 0.014, n = 3–5 per cohort for experiments). I) mRNA levels of lipid regulatory genes at 48 h after PH (P = 0.011, 0.014 and 0.037 respectively for PPARα, PPARδ and FATP5 but is otherwise non significant). n = 4 for *Fgl1^+/+^* except FATP5 where n = 3 per group. n = 5 for *Fgl1^−/−^*.

To initiate studies on the physiological functions of Fgl1, we generated a mouse null for Fgl1. The *Fgl1* gene comprises 8 exons spanning more than 24 kb of genomic DNA. The initiator ATG resides in the second exon. To disrupt *Fgl1*, we deleted exons 2 and 3 which removed the initiator ATG and abolished the secretory signal recognition peptide (Figure S1A in File S1). Genotyping confirmed the insertion of the neomycin cassette in the *Fgl1^−/−^* mouse (Figure S1B in File S1). To ascertain whether Fgl1 was absent in the knockout mouse, we performed qPCR assays and found that Fgl1 mRNA and protein were absent in the livers of *Fgl1^−/−^* mice at baseline and after PH (Figure 2A and B). We also observed that Fgl1 mRNA was absent from both brown and white adipose tissues of the *Fgl1^−/−^* mice (Figure S2 in File S1). Despite the loss of Fgl1 expression, *Fgl1^−/−^* mice appeared grossly normal when compared to wild type mates except that they were persistently heavier. This difference in size was obvious at 3 weeks, the earliest we could genotype our mice, at which point the Fgl1 null mouse were 37% heavier than wild type mates (Figure 2C, P<0.0001). We monitored a group of mice beginning at 7 weeks and find that the size difference remains as the mice age (Figure 2D). Indeed in a small cohort of mice we kept for 11 months this difference persisted (not shown). Considering Fgl1’s presumptive role in liver proliferation, we carefully examined liver tissue, but observed no differences between the livers of *Fgl1^+/+^* and *Fgl1^−/−^* mice by: i) liver weight (1.15+/−0.28 grams versus 1.03+/−0.05 grams respectively, Figure 2E), ii) histologic analysis by hematoxylin and eosin staining (not shown), iii) immunohistochemistry for PCNA and BRDU incorporation (not shown). However we found that the liver weight to body weight ratio, a quotient used to determine appropriate liver size for body weight [24] is smaller in *Fgl1^−/−^* when compared to wild type mates (Figure 2F, P = 0.0081). These findings confirm that the Fgl1 mice were not heavier as a result of increased liver size. At 48 h after PH, *Fgl1^−/−^* livers appeared grossly steatotic (Figure 2G, top panels). Histologic analysis demonstrated the presence of marked macrovesicular steatosis following PH at 48 h (Figure 2E, compare middle panel images) with resolution by 96 h (Figure 2G, compare right middle panel to right lower panel). In addition, lipid analysis showed that the triglyceride (TG) content of liver extract was markedly elevated at 48 h post PH (Figure 2H, P = 0.014). This suggests that Fgl1 has lipid regulatory roles that became manifest during recovery from liver injury. We next determined the expression levels of hepatic lipid regulatory genes; lipolysis: hepatic lipase (HL), oxidation: peroxisome proliferator-activated receptor alpha (PPARα, PPARγ1, PPARγ2, PPARδ and carnitine palmitoyltransferase (CPT1)), fatty acid transport genes: cluster of differentiation 36 (CD36), fatty acid transport protein 2 (FATP2) and FATP5 and lipid synthetic genes: fatty acid synthase (FAS) and acetyl-CoA carboxylase (ACC) at baseline and at 48 h after PH. With the exception of PPARδ (P = 0.016) which is upregulated in the *Fgl1^−/−^* liver, we found no differences in the expression of these cohort of genes at baseline (Figure S3 in File S1). At 48 h post PH however, we see a persistent difference in the expression of PPARδ (P = 0.011) in addition to enhanced expression of another lipid oxidation gene PPARα (P = 0.014) and lipid transport protein, FATP5 (P = 0.037) in the *Fgl1^−/−^* mouse (Figure 2I). These data suggest that Fgl1 regulates a pathway involved in hepatic lipid metabolism and that with its loss, lipid regulatory differences become phenotypically obvious during liver regeneration.

### Decreased Plasma Cholesterol and Free Fatty Acid Levels in the *Fgl1^−/−^* Mouse

**Figure 3:**
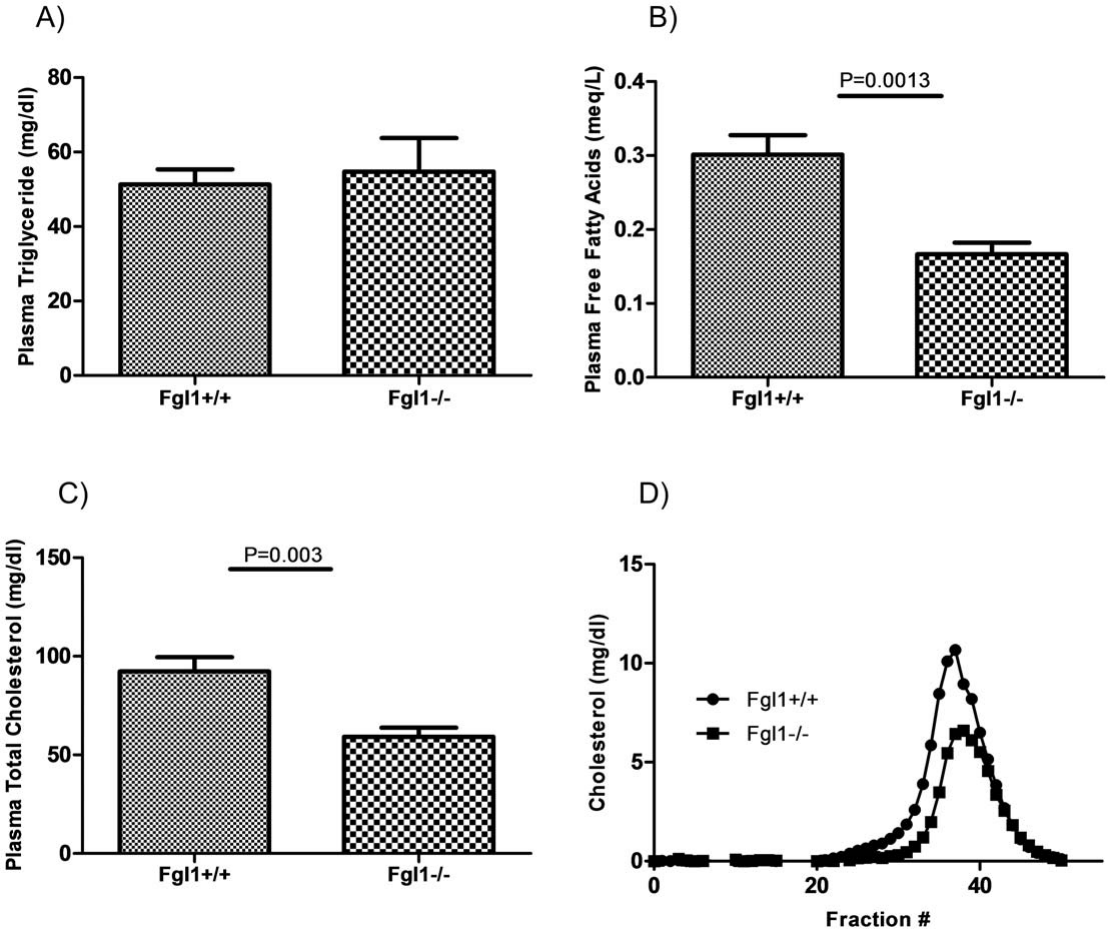
Plasma lipid, cholesterol and free fatty acid levels in the Fgl1 null mouse. A) There is no significant difference in steady state plasma TG levels of *Fgl1^+/+^* and *Fgl1^−/−^* mice. B) Free fatty acid levels are decreased in the Fgl1 null mouse (P = 0.001). C and D) Plasma cholesterol levels are levels are lower as determined by total cholesterol (P = 0.003) and FPLC analysis. n = 5 per group.

Considering the phenotype of the *Fgl1^−/−^* mice, the structural similarity between FGL1 and ANGPTL3 and 4 and the role of these proteins in regulating plasma lipids, we ascertained the lipid profile in the liver and plasma of fasted wild type and Fgl1 null mice. Hepatic concentrations of triglycerides (Figure 2G, left side bars), cholesterol and free fatty acids (not shown) were not significantly different in chow fed mice. In addition, plasma levels of plasma triglycerides were similar between the wild type and mutant mice (Figure 3A). However, plasma free fatty acid and cholesterol concentrations were decreased in the *Fgl1^−/−^* mice (Figure 3B and 3C, P = 0.001 and 0.003 respectively). There were no clear genotype-dependent differences in the VLDL containing fraction (Figure 3D), but HDL cholesterol content in the *Fgl1^−/−^* mice showed a decrease that was consistent with the reductions in total plasma cholesterol shown in Figure 3C. These data are in keeping with findings in mice with targeted disruption of *Angptl3* and *Angptl4* where in the fasted state triglyceride levels were unchanged but cholesterol levels were decreased [11].

### Fasting Hyperglycemia in the *Fgl1^−/−^* Mouse

**Figure 4:**
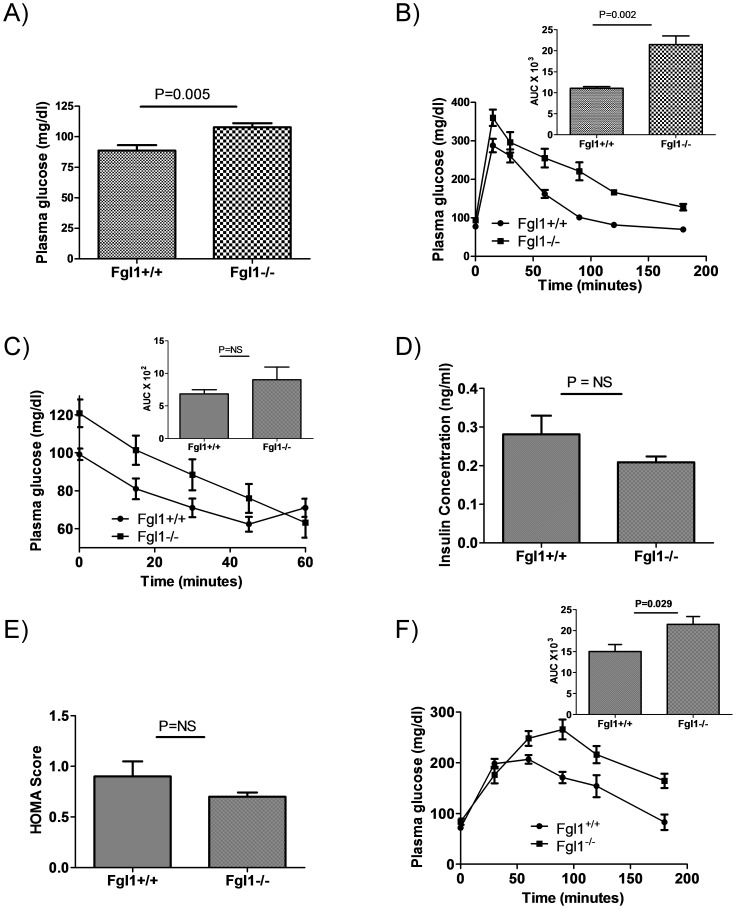
Glucose metabolism in the Fgl1 null mouse. A) *Fgl1^−/−^* mice have fasting hyperglycemia (P = 0.005, n = 10 for *Fgl1^+/+^* and n = 8 for *Fgl1^−/−^*). B) Glucose tolerance tests of fasted 3 month old mouse. The graph represents a plot of plasma glucose versus time after i.p. administration of glucose. The superimposed panel represents plots of average area under the curve (AUC) for each mouse. The baseline was set as the mean pre-glucose administration plasma level. The difference is glucose levels as determined from the AUC is significant (P = 0.002, n = 4 per group). C) Insulin tolerance test on 3 month old fasted mouse shows a similar rate of decline of glucose levels between the *Fgl1^+/+^* and *Fgl1^−/−^* mice. AUC calculations after normalizing for baseline glucose for the first 45 min of the test shows no differences between Fgl1 containing and deficient mice (n = 5 per group). D) Insulin levels are not different between *Fgl1^+/+^* and *Fgl1^−/−^* mice. E) HOMA scores are not different for *Fgl1^+/+^* and *Fgl1^−/−^* (n = 5 for *Fgl1^+/+^* and 4 for *Fgl1^−/−^*). F) Graph of glucose levels following administration of pyruvate to fasted mice. Inset is AUC calculation which shows a difference in glucose levels over the duration of the experiment (P = 0.029, n = 5 per group).

We next asked whether there were differences in glucose metabolism between the *Fgl1^+/+^* and *Fgl1^−/−^* mice. First we determined blood glucose after an overnight fast and found more profound fasting hyperglycemia in the *Fgl1^−/−^* mice (Figure 4A, P = 0.005) at 4 months of age. We next subjected these mice to glucose tolerance tests (GTT). *Fgl1^−/−^* mice exhibited reduced glucose tolerance, as evidenced by more profound elevation in plasma glucose concentrations. By 60 min after administration of glucose there was already marked difference in plasma glucose levels between the wild type and the mutant mice (Figure 4B). We determined the area under the curve (AUC) for each animal using basal levels of glucose and the average AUC for each *Fgl1^+/+^* and *Fgl1^−/−^* mice and found statistically significant differences in response to the administered glucose load (Figure 4B and superimposed, P = 0.002). We next asked whether these effects on glucose were due to differences in sensitivity to insulin and or due to differences in plasma insulin levels. We performed insulin tolerance tests (ITT) following an overnight fast on 4 month old mice. We found a similar magnitude in the reduction of glucose levels after exogenous administration of insulin, suggesting that both *Fgl1^+/+^*and *Fgl1^−/−^* mice were equally sensitive to administered insulin (Figure 4C). In support of this, we determined the area under the curve for each mouse and found no significant difference between the wild type and the Fgl1 null mouse (Figure 4C, superimposed). In addition, we measured insulin levels and found no differences (Figure 4D) and estimates of insulin sensitivity by homesostatic model assessment (HOMA) showed no differences (Figure 4E). These data suggest that hyperglycemia and defects in GTT were due to a non insulin dependent process. We next performed pyruvate tolerance test to ascertain if differences in gluconeogensis may account for the increased glucose levels in the *Fgl1^−/−^* mice. Figure 4F shows that *Fgl1^−/−^* mice have enhanced glucose synthesis when compared to wild type mates. The difference between the two cohorts is significant as determined by the AUC (Figure 4F, inset, P = 0.029).

### *Fgl1^−/−^* Mice Exhibit Structural and Functional Defects in Adipose Tissues

**Figure 5:**
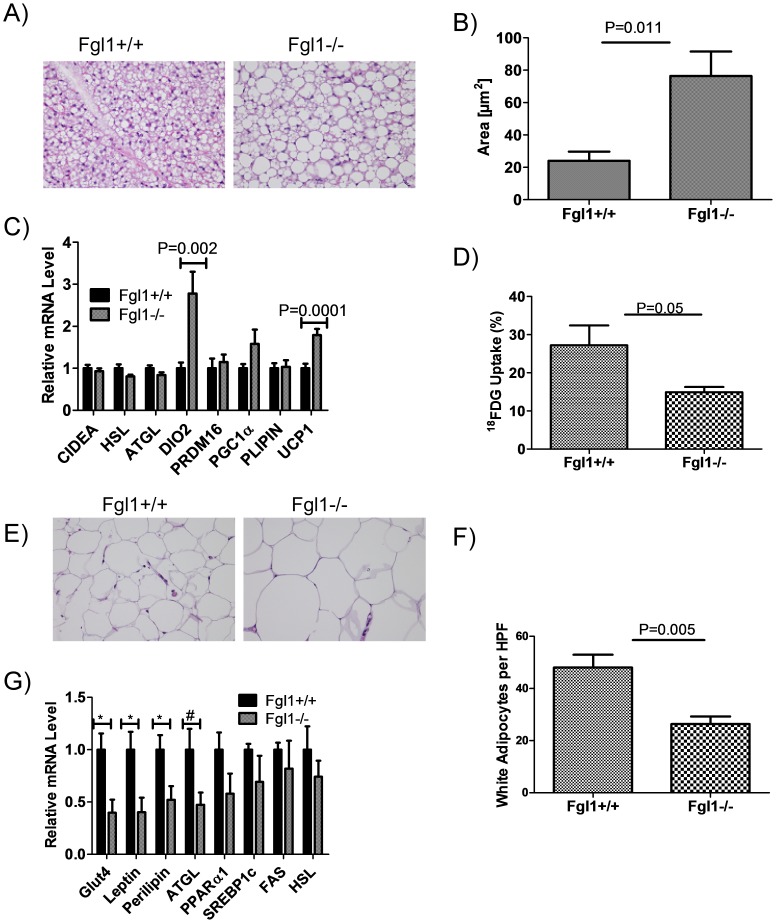
Structure, content and activity of adipose tissues in the Fgl1 null mouse. A) Representative H&E stains (40× magnification) of brown adipose tissue. Lipid droplets are larger in *Fgl1^−/−^* mice. B) Quantitation of lipid droplet size show significant difference between *Fgl1^+/+^* and *Fgl1^−/−^* mice (P = 0.011, n = 5 per group). C) Expression of brown adipose genes. Note the paradoxical up regulation of DiO2 and UCP1 (P = 0.002 and 0.0001 respectively. n = 11 per group except for Perilipin and HSL where n = 5 and 6 respectively). D) ^18^FDG incorporation into BAT. The % uptake represents the uptake of injected dose per gram of tissue. Note the marked decrease in radioisotope uptake in BAT (P = 0.05, n = 5 per group). E). Representative H&E stains (40× magnification) of white adipose tissue. Lipid droplets are larger in *Fgl1^−/−^* mice. F) Quantitation of number of cells per HPF shows smaller number of white adipose cells in *Fgl1^−/−^* (P = 0.005, n = 5). G) Expression of white adipose genes. Glut4, leptin and perilipin are significantly down regulated (*) with a P<0.04 for each. P for ATGL (#) is 0.06. n = 4–6 mice per group.

The difference in body weights between the *Fgl1^+/+^* and *Fgl1^−/−^* mice is not due to an enlarged liver (Figure 2E and 2F). To ascertain if increase in the weight of any organ accounted for the difference in size we determined the weight of the quadriceps muscle, cardiac muscle, inguinal white adipose tissue and brown adipose tissue. We also determined the length of each mouse from the nose to the anus. We found that there is a statistically significant difference in the length of the mice with the *Fgl1^−/−^* mouse longer than the *Fgl1^+/+^* mouse (93.40+/−0.40 millimeters versus 89.60+/−0.68 respectively, P = 0.001, Table 1). We also found more white adipose tissue content in the *Fgl1^−/−^* mouse (0.55 grams +/−0.11 versus 0.24+/−0.03 grams, P = 0.021, Table 1) which persists even after accounting for difference in body weight. These data imply that the *Fgl1^−/−^* mice are longer and fatter. Considering these data, we next examined white and brown adipose tissues by hemotoxylin and eosin staining and found differences between brown and white adipose tissue morphology of *Fgl1^+/+^*and *Fgl1^−/−^* mice. In mice lacking Fgl1, we found that the brown adipose tissue contained larger lipid droplets and had markedly reduced number of nuclei while the wild type mice had small sized fat particles more typical of brown fat (Figure 5A). Quantitative estimates confirm that the droplets in the *Fgl1^−/−^* mice are larger (Figure 5B, P = 0.011). We performed qPCR assays to ascertain differences in lipogenic gene expression between *Fgl1^+/+^* and *Fgl1^−/−^* mice. We found no differences in the level of brown adipocyte differentiation genes; PR domain containing 16 (PRDM16) and peroxisome proliferator-activated receptor gamma co-receptor 1α (PGC1α) or in lipolysis regulatory genes; hormone sensitive lipase (HSL), adipose triglyceride lipase (ATGL), cell death inducing DFFA-like effector a (CIDEA) and perilipin. Surprisingly, we find paradoxical up regulation of uncoupling protein 1(UCP1) and iodothyronine deiodinase type II (DiO2) in the brown adipose tissue of the *Fgl1^−/−^* mouse (Figure 5C, P = 0.0001 and 0.002 respectively). To elucidate functional defects in brown adipose tissue of *Fgl1^−/−^* mice, we administered ^18^FDG into mice and measured uptake by scintillation counting. We found a marked decrease in ^18^FDG in brown fat tissue of *Fgl1^−/−^* mice (Figure 5D, P = 0.05). This suggests a defect at the level of brown fat tissue either in its ability to efficiently take up glucose or in its activity. In white adipose tissue of the Fgl1 null mouse, we found larger fat containing cells when compared *Fgl1^+/+^* mice (Figure 5E), supported by the a lower number of white adipocytes per high power field in white adipose tissue of the *Fgl1^−/−^* mouse (Figure 5F, P = 0.005). We analyzed white adipose tissue to ascertain differences in the expression of regulatory genes. We found no significant differences in the expression PPARα, sterol regulatory element binding protein 1c (SREBP1c), fatty acid synthase (FAS) and HSL. We however find suppression of Glut4, leptin and perilipin (Figure 5G, P<0.04 for all three genes). These results suggest that there are structural, molecular and functional differences in adipose tissue of mice that express or lack Fgl1.

**Table 1:** Length and tissue mass of *Fgl1^+/+^* and *Fgl1^−/−^* mice.

	*Fgl1^+/+^*	*Fgl1^−/−^*	P value
Weight (grams)	21.91+/−0.49	25.26+/−1.27	0.039
Length (millimeters)	89.60+/−0.68	93.40+/−0.40	0.001
Liver (grams)*	1.15+/−0.03	1.03+/−0.05	NS
Heart (grams)	0.11+/−0.01	0.14+/−0.01	NS
Quadriceps muscle(grams)	0.19+/−0.01	0.21+/−0.03	NS
White adipose (grams)	0.24+/−0.03	0.55+/−0.11	0.021
Brown adipose (grams)	0.06+/−0.01	0.09+0.01	NS

(n = 5 for both cohorts except for liver (*) where n = 9).

### Food Consumption is Similar between *Fgl1^+/+^* and *Fgl1^−/−^* Mice

**Figure 6:**
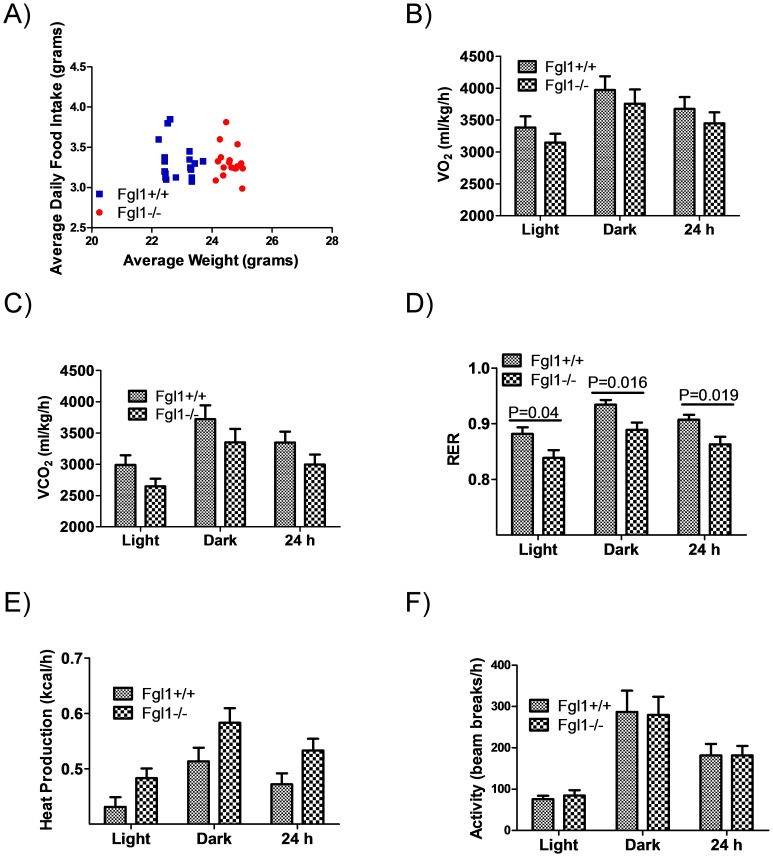
Food intake and indirect calorimetry. Scatter plot of average food intake versus average weight for individually housed *Fgl1^+/+^* (blue squares, n = 4) and *Fgl1^−/−^*(red circles, n = 8) taken daily over an 18 day period. Note that *Fgl1^−/−^* remain larger that wild types for the duration of the experiment. B and C) Indirect calorimetric values for VO_2_ and VCO_2_ respectively and D) RER. The RER is significant irrespective of day and night cycles (P = 0.04 and 0.016 respectively) and over the entire 24 h (P = 0.019). E) Heat generation is not significantly different and F) activity is not different between the Fgl1 containing and deficient mice. n = 6 for Figures 6B to 6F.

Our *Fgl1^−/−^* mice are heavier and thus we wondered if the increased size was due to increased food consumption. We determined food consumption over an 18 day period for individually housed mice by measuring daily food intake and daily weights. Figure 6A shows that *Fgl1^−/−^* were heavier than wild type mates through out the experiment. However, mean food consumption per day was similar for both groups (3.32+/−0.04 g versus 3.29+/−0.04 g for *Fgl1^+/+^*and *Fgl1^−/−^* respectively). Because there were multiple observations per individual mouse, we performed mixed regression analysis (mixed effect models the weight of subjects based upon the number of measurements and their variability, and thus yield more appropriate standard errors than linear regression). We used food intake as the dependent variable and genotype as the independent variable, adjusted for body weight at each measurement day. As expected, we found no relationship between genotype and daily food intake (beta coefficient for mutant versus wild type mice (−0.088 g food, P = 0.21)). These data suggest that increased food intake is not the reason for the larger size of the *Fgl1^−/−^*mice. To determine whether the *Fgl1^−/−^* and *Fgl1^+/+^* mice differed metabolically, we used indirect calorimetry to determine oxygen consumption (VO_2_), carbon dioxide generation (VCO_2_), the respiratory quotient (RER) and heat exchange. We also determined activity for each cohort. We found no differences in activity between the wild type and Fgl1 null mouse (Figure 6F). We found statistically insignificant but lower absolute values lower VO_2_ and VCO_2_ for the *Fgl1^−/−^* mice irrespective of time of day (Figure 6B and C). However, we found a significantly lower RER in the *Fgl1^−/−^* mice during day light h, night time h and for the entire 24 h period (Figure 6D, P = 0.04, P = 0.016 and P = 0.019 respectively). These data suggest that Fgl1 null mice were more dependent on fatty oxidation for energy source when compared to the wild type mates. We used the indirect calorimetric data to determine the heat generation for each cohort and found no significant differences between the two cohorts (Figure 6E).

## Discussion

This study was designed to elucidate the functions of Fgl1, a primarily liver secreted protein with mitogenic activity on hepatocytes. We establish that Fgl1 has novel functions in glucose metabolism and energy substrate utilization. The major findings are: i) Fgl1was expressed in low levels in adipose tissue but that this expression is augmented following liver injury ii) Fgl1 null mice had lower fasting plasma concentrations of cholesterol and free fatty acids iii) Fgl1 null mice exhibited fasting hyperglycemia and enhanced hepatic glucose production iv) Fgl1 null mice were heavier than wild type mates despite consuming similar amounts of food.

Previous studies on Fgl1 have focused primarily on its role in hepatocyte biology, showing that the protein is secreted by hepatocytes, enhances tritiated thymidine incorporation into hepatocytes, and rescues animals from liver failure [3], [4], [6]. In the absence of liver injury, deletion of Fgl1 did not lead to any gross hepatic defects, whereas following injury to the liver, there were marked increases in hepatic triglyceride accumulation in the Fgl1 null mouse, suggesting that metabolic stress may have revealed this novel biological function of Fgl1. This was supported by the observation that Fgl1 expression in brown adipose tissue is enhanced following PH, similar in magnitude to its induction in the liver following PH. These findings are in keeping with the observations of Simek and colleagues from almost half a century ago who suggested a crosstalk between adipose tissue and the liver through regulated lipolysis during liver regeneration [25]. Whereas the molecular mechanisms that underlie cross-talk between liver and adipose tissue during liver regeneration remain largely unknown, the profound hepatic steatosis observed in the Fgl1 null mice during regeneration suggests a key regulatory role for Fgl1 in linking liver regeneration with adipose tissue function.

In the absence of Fgl1, we found decreased cholesterol and free fatty acid concentrations. This is in keeping with observations from the natural loss of Angptl3 in the KK/San mouse showing decreased cholesterol and free fatty acids [10], [16] and the with targeted disruption of *Angptl3* and *Angptl4* which also exhibit low plasma cholesterol levels [11]. Thus, loss of Fgl1 phenocopies plasma lipid concentrations in mice devoid of *Angptl3* and *Angptl4.* These effects are believed to be mediated by the ability of both ANGPTL proteins to inhibit clearance of VLDL and chylomicron triglycerides into peripheral tissues or through stimulation of adipose tissue lipolysis resulting in increased free fatty acid levels and triglycerides [10], [15], [16]. In this regard, we are particularly intrigued by the fact that adipocytes from brown and white adipose tissue of the *Fgl1^−/−^* mice have comparably larger sized fat containing cells. We also found decreased mRNA levels of the lipolytic enzyme ATGL, in white adipose tissue. These data suggest that impairment in adipose tissue lipolysis may be the principal culprit that leads to decreased plasma free fatty acid concentrations. In addition, the accumulation of triglycerides in the liver of the Fgl1 null mouse during recovery from liver injury and the differential regulation of FATP5 which enhances transport of fatty acids into the liver and of PPARα and δ, two factors whose activity are regulated by products of lipolysis [26], [27], [28] support the notion that Fgl1 may regulate hepatic lipid concentrations during liver regeneration.

Mice deficient of Fgl1 have fasting hyperglycemia, similar insulin sensitivity and enhanced gluconeogenesis. We suggest that these impairments likely result from physiologic differences in both hepatic and adipose tissues. Because insulin levels and response to exogenous insulin administration are similar, we speculate that enhanced glucose production occurs through an insulin independent mechanism. Because adipocytes have been implicated in the regulation of glucose homeostasis, our finding of defects in their structure and in gene expression suggests that they may contribute to the differences in glucose levels between the *Fgl1^+/+^* and *Fgl1^−/−^* mice. In brown fat, we find decreased ^18^FDG uptake suggesting that glucose uptake into this adipose tissue is also reduced. In white adipose tissue, we found significant down regulation of Glut4, a gene whose loss in adipose tissue results in hyperglycemia and impaired glucose handling [29], [30]; leptin a gene whose product enhances peripheral glucose uptake [31] and perilipin, a white adipose protein whose over expression protects from diet induced obesity and improves glucose tolerance [32]. Thus the hyperglycemia in the Fgl1 null mouse likely occurs as a result of increased hepatic production and decreased uptake into adipose tissues.

Loss of Fgl1 results in an increase in size of the mice despite similar food consumption. However, quantitative estimates of tissue mass show that *Fgl1^−/−^* mouse have larger white adipose tissue mass. We believe that the increased white adipose content likely accounts for some of the difference in size and suggests that the *Fgl1^−/−^* mouse is fatter than it wild type counterpart. In addition, we speculate that the *Fgl1^−/−^* mice have defects in brown adipose function and may be hypometabolic because 1) they eat the same as wild type mates despite a larger size 2) they have morphologically more white adipose-like lipid droplets 3) they have reduced uptake of ^18^ FDG, a marker for brown fat function 4) they have paradoxical upregulation of DiO2, an enzyme that regulates brown adipose thyroid hormone levels and 5) of UCP1 a canonical brown adipose protein and a biochemical marker of brown adipose activation. The decreased ^18^FDG uptake in the absence of differences insulin levels suggest an insulin independent uptake of glucose into brown fat, typical of this tissue [33], [34] and implies a defect in brown adipose tissue function at this level. In addition the paradoxical upregulation of UCP1 transcripts suggest that Fgl1 may help regulate appropriate activation of UCP1 and that in its absence transcript levels are regulated as an adaptive response. Lastly, published data suggest that loss of DiO2 results intra adipose hypothyroidism, despite systemic euthyroid state [35] and we speculate that the paradoxical upregulation of DiO2 mRNA levels suggests a defect in brown adipose tissue function.

A number of issues raised by the studies presented remain unanswered. Firstly, previous studies have suggested that Fgl1 acts as a paracrine factor on hepatocytes [9]. However we have previously shown that Fgl1 is present in the plasma [4] suggesting that it may have hormonal action on distant targets. The current studies show that Fgl1 transcript is present in low levels in adipose tissues implying that that low levels of protein secreted by adipose tissues could have paracrine effects. Regardless, we are cognizant of the fact that adipose tissues may also be hormonally affected by hepatic derived Fgl1. Studies on the specific effect of Fgl1 on adipose tissue will have to await the identification of a receptor and/or through tissue specific ablation of Fgl1. Alternatively, *in vitro* studies of brown adipocyte differentiated cells from *Fgl1^+/+^* and *Fgl1^−/−^* mice may yield insight into the exact role of Fgl1 in this adipose tissue. Secondly, the mechanisms governing hepatic steatosis during liver regeneration in the *Fgl1^−/−^* mice remain incompletely defined. Our results suggest that there is increased fatty acid transport into hepatocytes likely mediated by FATP5. However, there is concurrent increase in PPARα and PPARδ levels which would be expected to result in enhanced oxidation of lipids and resolution of steatosis. We suspect that given that the steatosis is transient and resolved by 96 h after PH, the increase in oxidative gene expression may be a result of but not the cause of the steatosis. Future experiments with detailed time course analysis will allow us to identify the mechanisms that govern this hepatic defect. Thirdly, despite a higher white adipose content, *Fgl1^−/−^* mouse have lower RERs suggesting more dependence on fat for metabolism. This may be an artifact of stress response induced by independent housing of mice for these experiments and not reflective of free living condition or of group-housed animals. Indeed, we found that when mice were moved from a group housing to independent cages to determine food consumption shown in Figure 6A, they lost weight and were then sluggish with regards to increased weight during the subsequent 18 days. Although there was no significant difference in average weight loss between the two groups, it is possible that the stress of isolation leads to more dependence on fat based metabolism and to lower RER in the *Fgl1^−/−^*.

Taken together, our data suggests a previously unrecognized role for Fgl1 in regulation of intermediate metabolism. In addition, we propose Fgl1 as a new angiopoietin like factor given i) structural conservation between Fgl1 and ANGPTLs ii) a similar tissue expression with hepatic and adipose tissue expression iii) similar defects in plasma lipids levels with low free fatty acids and cholesterol in Fgl1 similar to *Angptl3* and *4* knockouts and iv) impaired glucose metabolism as seen in the *Angptl6* knockout.

## Supporting Information

File S1 **This file contains three supporting figures and one supporting table.** Figure S1: The Fgl1 null mouse. Figure S2: Fgl1 is present in BAT and WT of *Fgl1^+/+^* mice but absent in the *Fgl1* null mice. Figure S3: Expression of lipid regulatory genes in livers of *Fgl1^+/+^* and *Fgl1^−/−^* mice at baseline. Table S1: Primer sequences.
(DOC)
