# Supplementary material for: Targeted Deletion of Fibrinogen Like Protein 1 Reveals a Novel Role in Energy Substrate Utilization
Source: PLoS One. 2013 Mar 6;8(3):e58084. doi: 10.1371/journal.pone.0058084 (PMC3590190; doi:10.1371/journal.pone.0058084)
Supplement: File S1 — This file contains three supporting figures and one supporting table. Figure S1: The Fgl1 null mouse. Figure S2: Fgl1 is present in BAT and WT of Fgl1+/+ mice but absent in the Fgl1 null mice. Figure S3: Expression of lipid regulatory genes in livers of Fgl1+/+ and Fgl1−/− mice at baseline. Table S1: Primer sequences. (DOC) [file pone.0058084.s001.doc]

**Supporting Information**


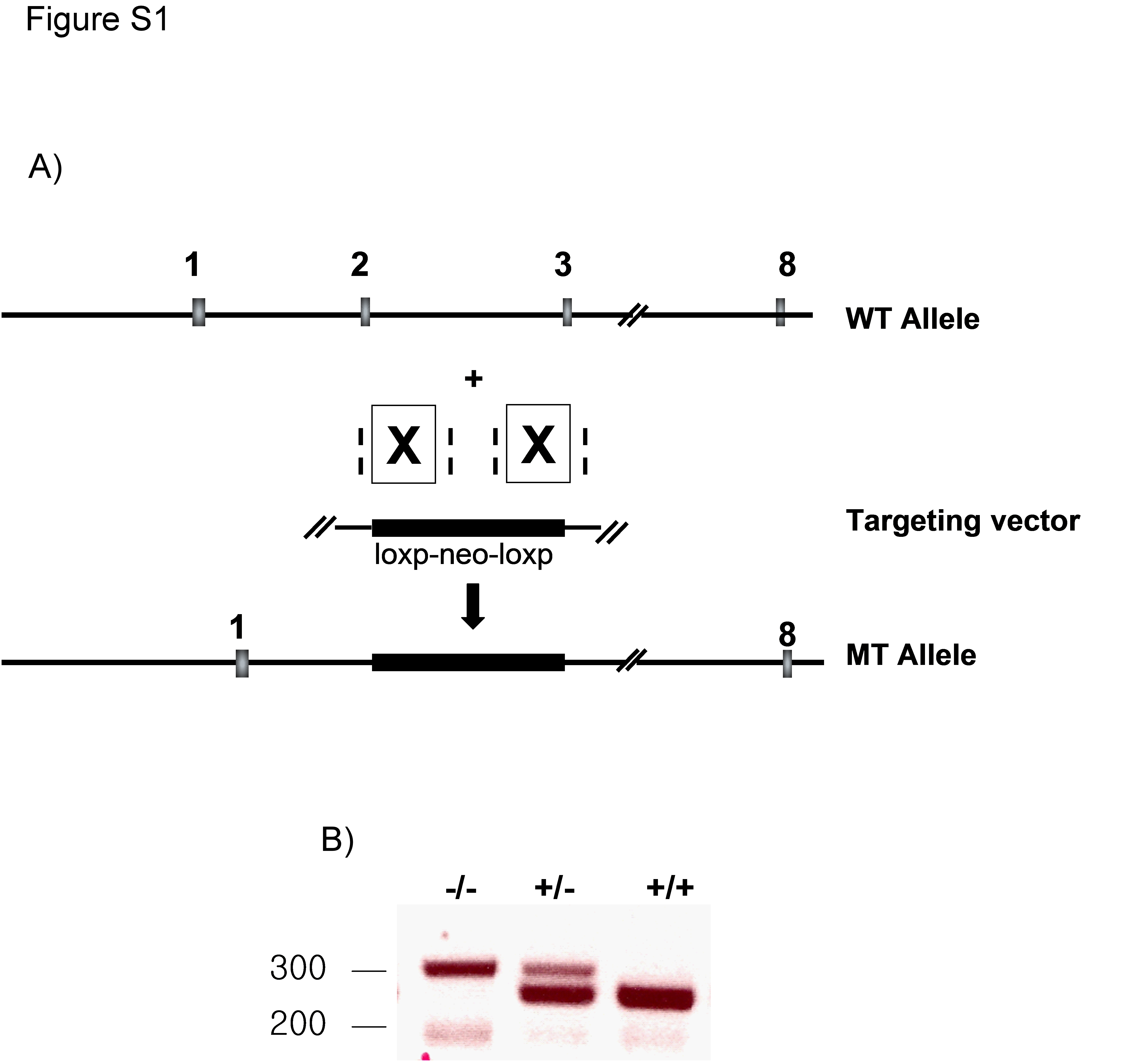


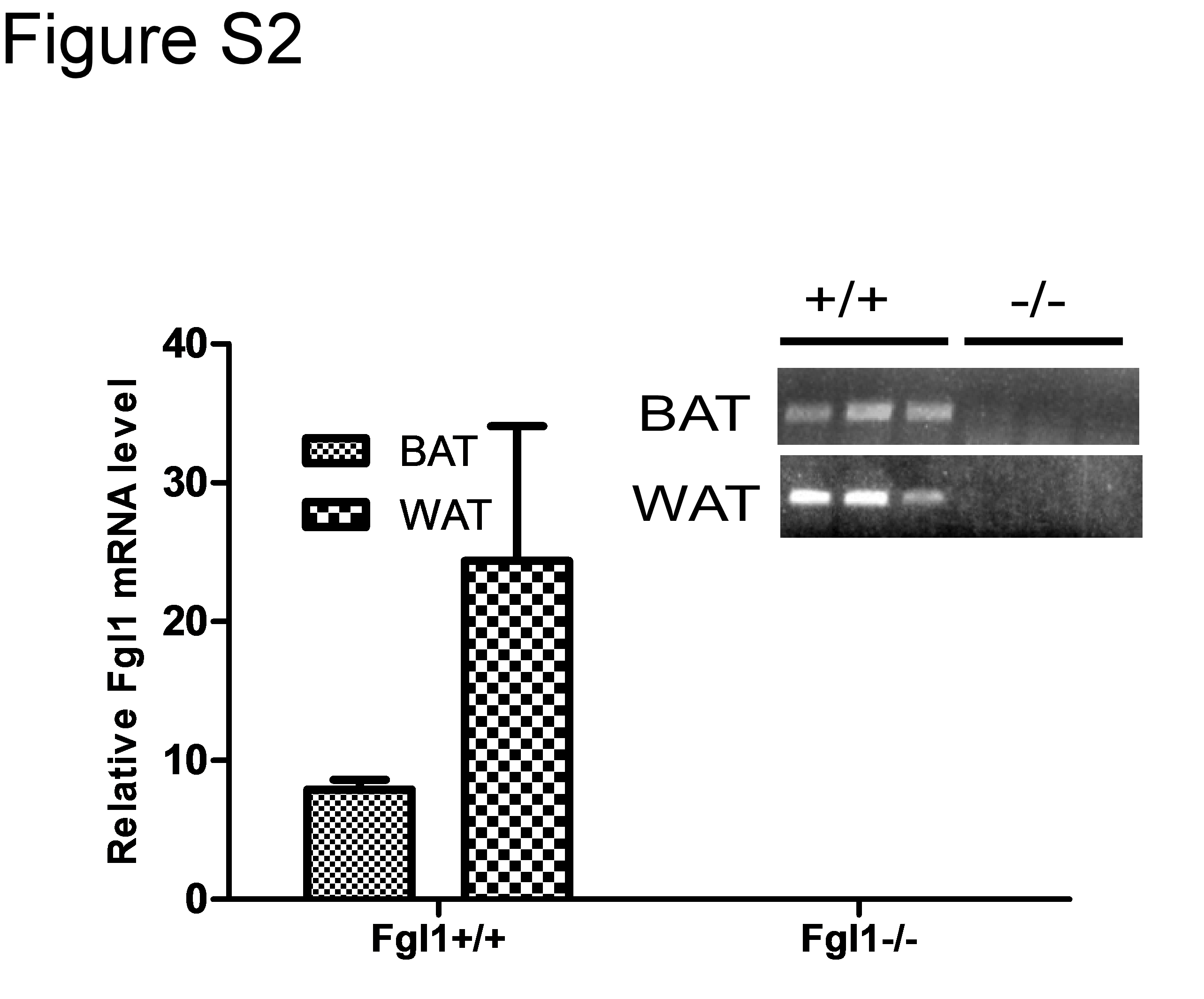


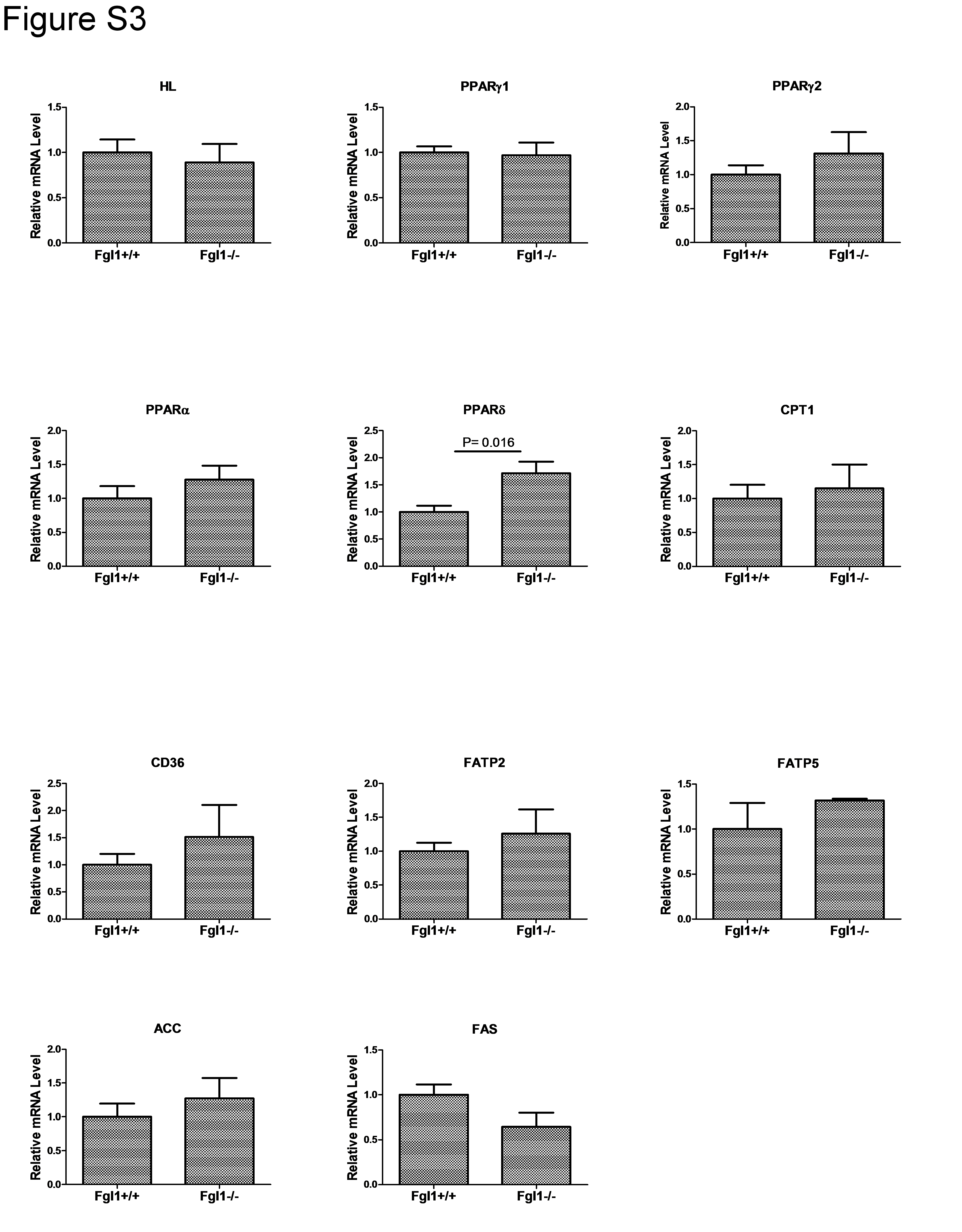


Table S1: Primer Sequences

| **Gene** | **Forward** | **Reverse** |
| --- | --- | --- |
| Fg1l | GATTTACAGCTTCGTCCTGG | TCTTGCCTCCAAGGTCAATG |
| Cyclophilin A | GGCCGATGACGAGC | TGTCTTTGGAACTTTGTCTGCAA |
| UCP1 | ACTGCCACACCTCCAGTCATT | CTTTGCCTCACTCAGGATTGG |
| Dio2 | GTCCGCAAATGACCCCTTT | CCCACCCACTCTCTGACTTTC |
| ATGL | TGATGACCACCCTTTCCAACA | GCAGAGTATAGGGCACCATCATG |
| PRDM16 | CAGCACGGTGAAGCCATTC | GCGTGCATCCGCTTGTG |
| Cidea | CCGAGTACTGGGCGATACAGA | GGTTACATGAACCAGCCTTTGG |
| HSL | TGTGGCTTGCGCTCTGG | CCGCGCGAACATGACC |
| SREBP1c | GGAGCCATGGATTGCACATT | GCTTCCAGAGAGGAGGCCAG |
| Perilipin | GCTCTTCAATACCCTCCAGAAAAG | TTCGAAGGCGGGTAGAGATG |
| Leptin | CACACACGCAGTCGGTATCC | GGTGAAGCCCAGGAATGAAG |
| FAS | AGGATGTCAACAAGCCCAAG | ACAGAGGAGAAGGCCACAAA |
| Glut4 | CCCGGACCCTATACCCTATT | GGCATTGATAACCCCAATGT |
| CPT1 | CCAATCATCTGGGTGCTGG | AAGAGACCCCGTAGCCATCA |
| FATP5 | GGAACTCTACGGCTCCACAG | GGCTCTGCCGTCTCTATGT |
| FATP2 | CTGCATGTCTTCTTGGAGCA | GCGTAGGTAAGCGTCTCGTC |
| CD36 | GGCTGTGTTTGGAGGCATTCT | CAAAAACTGGGTGAAAACGG |
| PPARα | CTACGGAGTTCACGCATGTG | TTGTCGTACACCAGCTTCAGC |
| PPARγ1 | GTGAACCACTGATATTCAGGA CAT TT | CCACAGAGCTGATTCCGAAGT |
| PPARδ | GCCATCATTCTGTGTGGAGAC | CCGTCTTCTTTAGCCACTGC |

**Supporting Information Legends**

Figure S1: The Fgl1 null mouse. A) Schematic representation of the targeting and disruption of the Fgl1 allele. The insertion of the loxp-neo-loxp disrupts the genomic structure and abolishes the initiating ATG and the signal recognition sequence which reside in exon 2. B) Genotyping using primers proximal to and distal to exon 2 detects the wild type allele (lane 3) while primers that detect sequences within the loxp-neo-loxp detect the disrupted allele (lane 1).

Figure S2: Fgl1 is present in BAT and WT of *Fgl1^+/+^* mice but absent in the *Fgl1* null mice. n=5 per cohort. Inset: agarose gel representation of PCR products of brown adipose tissue (BAT) and white adipose tissue (WAT) from *Fgl1^+/+^* ( +/+) and  *Fgl1^-/-^* ( -/-) mice. Note the absence of Fgl1 in adipose tissues of the knockout mouse. Cyclophilin A is the reference gene.

Figure S3: Expression of lipid regulatory genes in livers of *Fgl1^+/+^* and *Fgl1^-/-^* mice at baseline. Of note there is only significant enhancement of PPARδ in *Fgl1^-/-^* (P=0.016). The differences between the remaining genes are not significant.
